# Supplementary material for: MetaRibo-Seq measures translation in microbiomes
Source: Nat Commun. 2020 Jun 29;11:3268. doi: 10.1038/s41467-020-17081-z (PMC7324362; doi:10.1038/s41467-020-17081-z)
Supplement: Supplementary file 10 — Supplementary Data 7 [file 41467_2020_17081_MOESM10_ESM.zip › File2/Confidence_VeryHigh_Taxonomy/128312_out.krona.html]

Javascript must be enabled to view this page.

members
magnitude
magnitudeUnassigned
count
unassigned
taxon
rank

128312\_out

6

superkingdom
2
6

1239
6
phylum

6
186801
class

order
186802
6

family
541000
5

genus
1940255
2

1650663
2

SRS893378\_contig\_number\_14289SRS971275\_contig\_number\_contig-100\_15511.86436
species

1
2048137
genus


SRS147139\_contig\_number\_31984
species
39484
1


SRS147139\_contig\_number\_44686
species
2291991
1

1017280
1
genus

2292244
1

SRS148721\_contig\_number\_32196
species

family
186803
1

841
1

SRS148159\_contig\_number\_50131
genus
